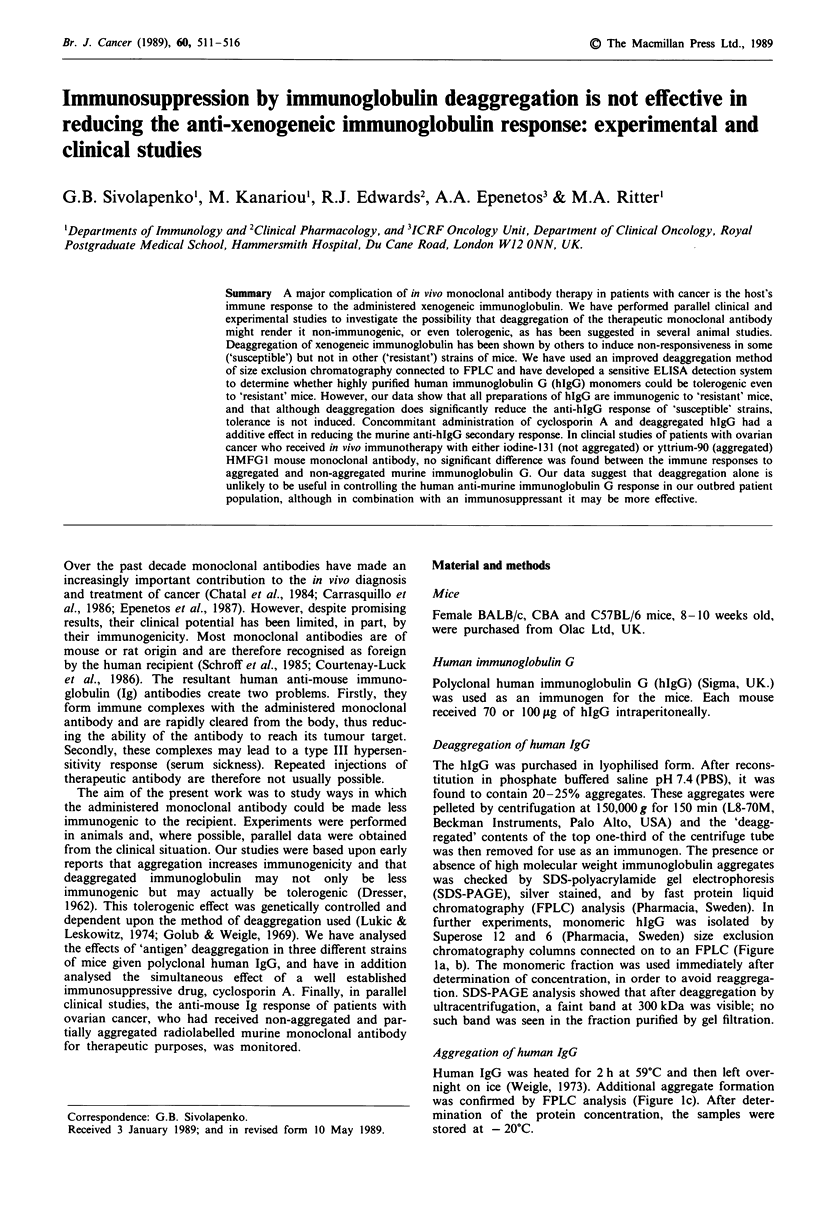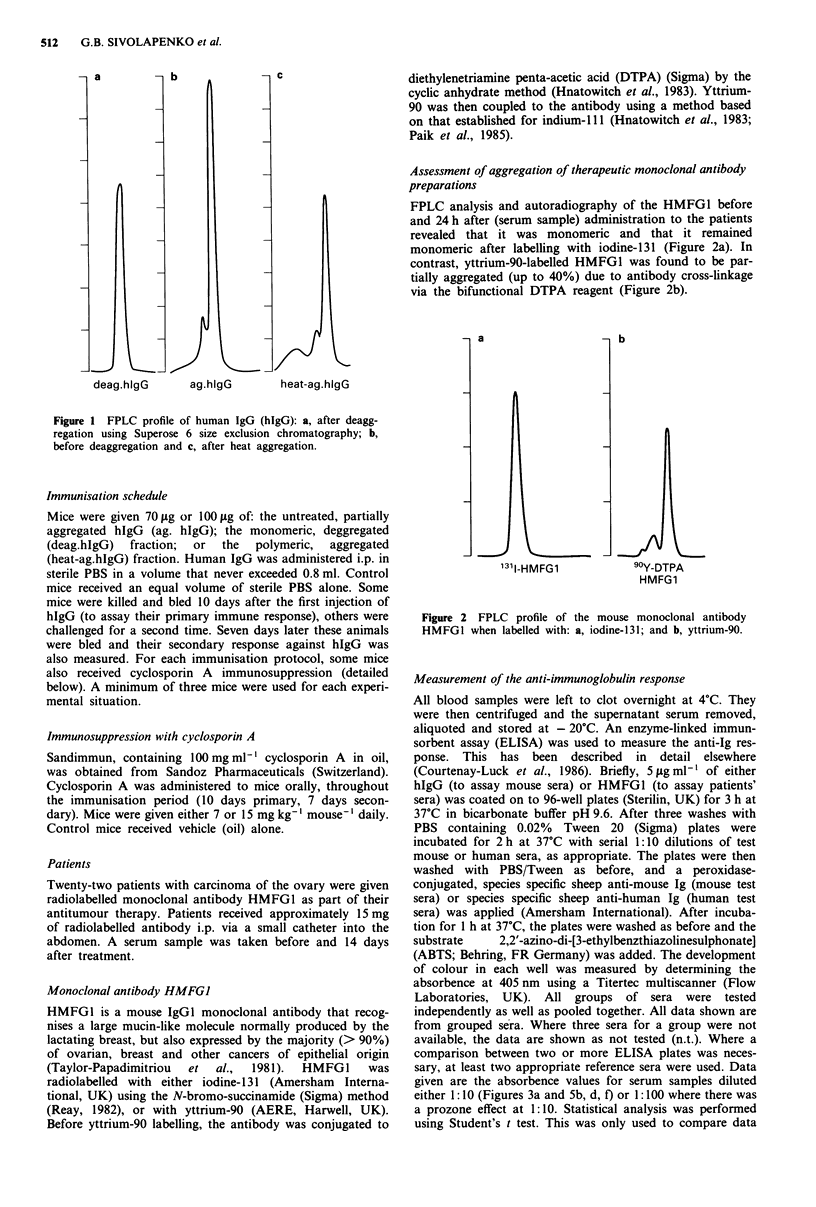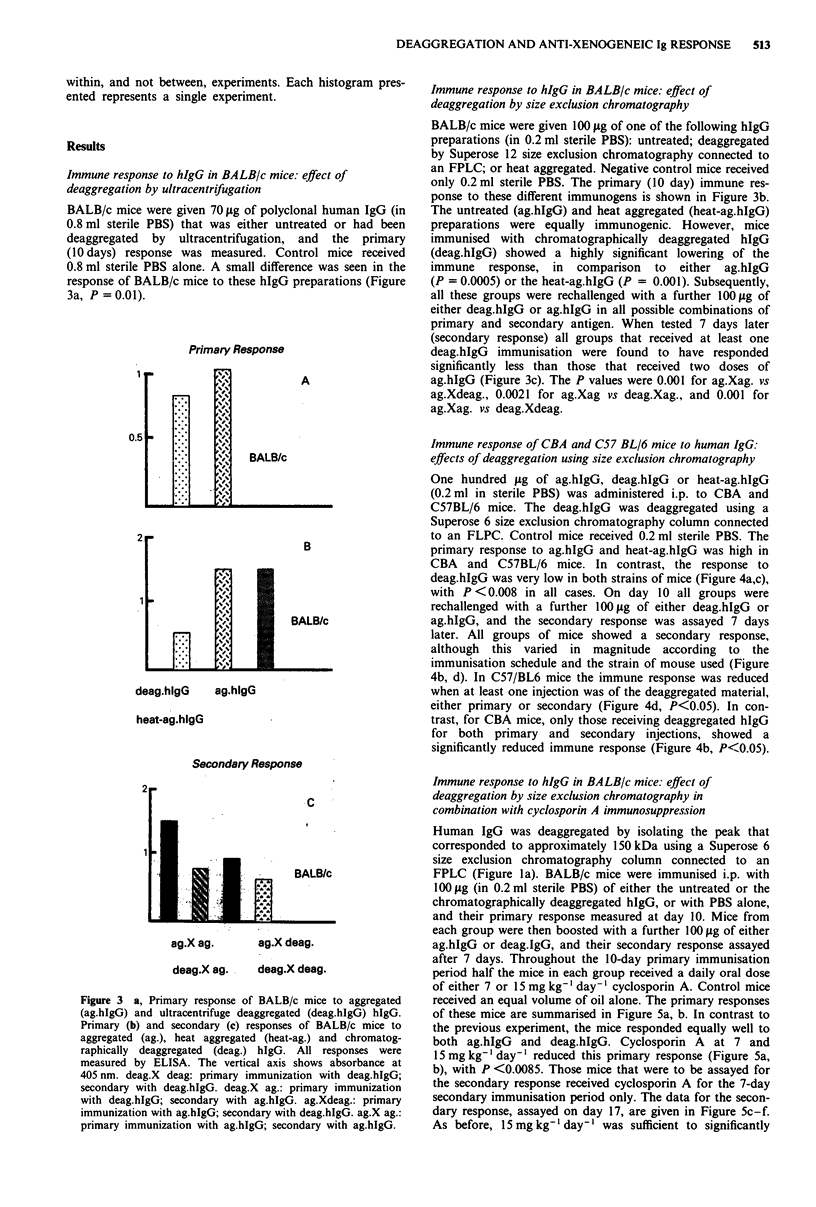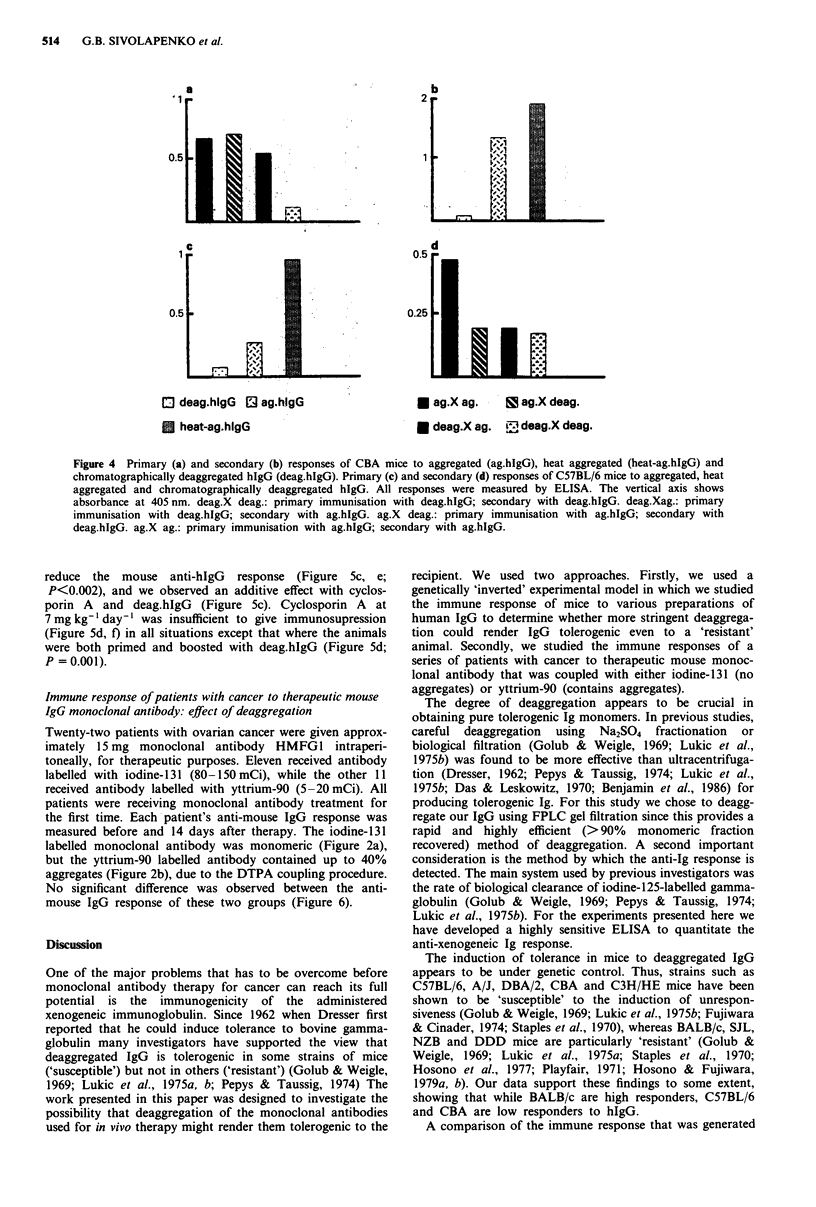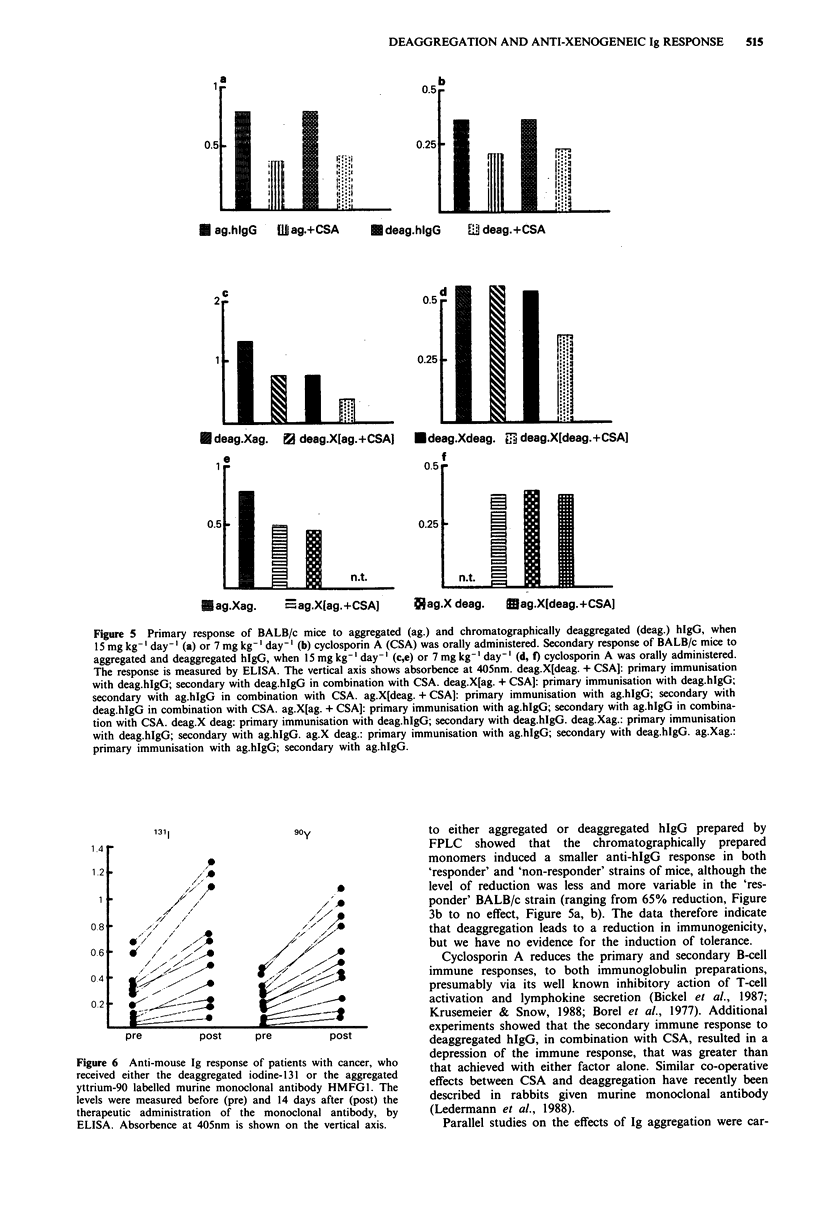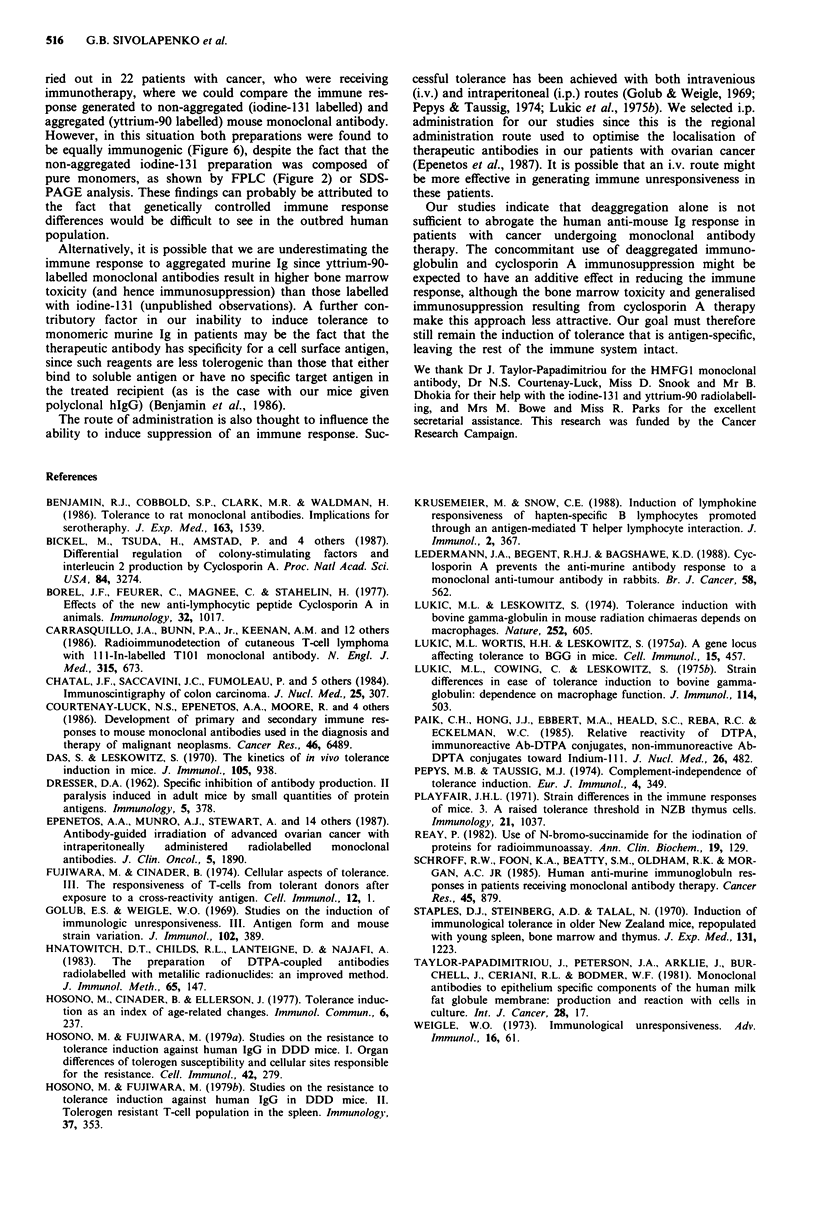# Immunosuppression by immunoglobulin deaggregation is not effective in reducing the anti-xenogeneic immunoglobulin response: experimental and clinical studies.

**DOI:** 10.1038/bjc.1989.304

**Published:** 1989-10

**Authors:** G. B. Sivolapenko, M. Kanariou, R. J. Edwards, A. A. Epenetos, M. A. Ritter

**Affiliations:** Department of Immunology, Royal Postgraduate Medical School, Hammersmith Hospital, London, UK.

## Abstract

A major complication of in vivo monoclonal antibody therapy in patients with cancer is the host's immune response to the administered xenogeneic immunoglobulin. We have performed parallel clinical and experimental studies to investigate the possibility that deaggregation of the therapeutic monoclonal antibody might render it non-immunogenic, or even tolerogenic, as has been suggested in several animal studies. Deaggregation of xenogeneic immunoglobulin has been shown by others to induce non-responsiveness in some ('susceptible') but not in other ('resistant') strains of mice. We have used an improved deaggregation method of size exclusion chromatography connected to FPLC and have developed a sensitive ELISA detection system to determine whether highly purified human immunoglobulin G (hIgG) monomers could be tolerogenic even to 'resistant' mice. However, our data show that all preparations of hIgG are immunogenic to 'resistant' mice, and that although deaggregation does significantly reduce the anti-hIgG response to 'susceptible' strains, tolerance is not induced. Concomitant administration of cyclosporin A and deaggregated hIgG had a additive effect in reducing the murine anti-hIgG secondary response. In clinical studies of patients with ovarian cancer who received in vivo immunotherapy with either iodine-131 (not aggregated) or yttrium-90 (aggregated) HMFG1 mouse monoclonal antibody, no significant difference was found between the immune responses to aggregated and non-aggregated murine immunoglobulin G. Our data suggest that deaggregation alone is unlikely to be useful in controlling the human anti-murine immunoglobulin G response in our outbred patient population, although in combination with an immunosuppressant it may be more effective.


Br. J. Cancer (1989), 60, 511-516 © The Macmillan Press Ltd., 1989

### Immunosuppression by immunoglobulin deaggregation is not effective in reducing the anti-xenogeneic immunoglobulin response: experimental and clinical studies

G.B. Sivolapenko[1], M. Kanariou[1], R.J. Edwards[2], A.A. Epenetos[3] & M.A. Ritter[1]

[1]*Departments of Immunology and* [2]*Clinical Pharmacology, and* [3]*ICRF Oncology Unit, Department of Clinical Oncology, Royal Postgraduate Medical School, Hammersmith Hospital, Du Cane Road, London W12 0NN, UK.*

**Summary** A major complication of *in vivo* monoclonal antibody therapy in patients with cancer is the host's immune response to the administered xenogeneic immunoglobulin. We have performed parallel clinical and experimental studies to investigate the possibility that deaggregation of the therapeutic monoclonal antibody might render it non-immunogenic, or even tolerogenic, as has been suggested in several animal studies. Deaggregation of xenogeneic immunoglobulin has been shown by others to induce non-responsiveness in some ('susceptible') but not in other ('resistant') strains of mice. We have used an improved deaggregation method of size exclusion chromatography connected to FPLC and have developed a sensitive ELISA detection system to determine whether highly purified human immunoglobulin G (hIgG) monomers could be tolerogenic even to 'resistant' mice. However, our data show that all preparations of hIgG are immunogenic to 'resistant' mice, and that although deaggregation does significantly reduce the anti-hIgG response of 'susceptible' strains, tolerance is not induced. Concommitant administration of cyclosporin A and deaggregated hIgG had a additive effect in reducing the murine anti-hIgG secondary response. In clincial studies of patients with ovarian cancer who received *in vivo* immunotherapy with either iodine-131 (not aggregated) or yttrium-90 (aggregated) HMFG1 mouse monoclonal antibody, no significant difference was found between the immune responses to aggregated and non-aggregated murine immunoglobulin G. Our data suggest that deaggregation alone is unlikely to be useful in controlling the human anti-murine immunoglobulin G response in our outbred patient population, although in combination with an immunosuppressant it may be more effective.

Over the past decade monoclonal antibodies have made an increasingly important contribution to the *in vivo* diagnosis and treatment of cancer (Chatal *et al.*, 1984; Carrasquillo *et al.*, 1986; Epenetos *et al.*, 1987). However, despite promising results, their clinical potential has been limited, in part, by their immunogenicity. Most monoclonal antibodies are of mouse or rat origin and are therefore recognised as foreign by the human recipient (Schroff *et al.*, 1985; Courtenay-Luck *et al.*, 1986). The resultant human anti-mouse immunoglobulin (Ig) antibodies create two problems. Firstly, they form immune complexes with the administered monoclonal antibody and are rapidly cleared from the body, thus reducing the ability of the antibody to reach its tumour target. Secondly, these complexes may lead to a type III hypersensitivity response (serum sickness). Repeated injections of therapeutic antibody are therefore not usually possible.

The aim of the present work was to study ways in which the administered monoclonal antibody could be made less immunogenic to the recipient. Experiments were performed in animals and, where possible, parallel data were obtained from the clinical situation. Our studies were based upon early reports that aggregation increases immunogenicity and that deaggregated immunoglobulin may not only be less immunogenic but may actually be tolerogenic (Dresser, 1962). This tolerogenic effect was genetically controlled and dependent upon the method of deaggregation used (Lukic & Leskowitz, 1974; Golub & Weigle, 1969). We have analysed the effects of 'antigen' deaggregation in three different strains of mice given polyclonal human IgG, and have in addition analysed the simultaneous effect of a well established immunosuppressive drug, cyclosporin A. Finally, in parallel clinical studies, the anti-mouse Ig response of patients with ovarian cancer, who had received non-aggregated and partially aggregated radiolabelled murine monoclonal antibody for therapeutic purposes, was monitored.

Correspondence: G.B. Sivolapenko.
Received 3 January 1989; and in revised form 10 May 1989.

### Material and methods

### *Mice*

Female BALB/c, CBA and C57BL/6 mice, 8-10 weeks old, were purchased from Olac Ltd, UK.

### *Human immunoglobulin G*

Polyclonal human immunoglobulin G (hIgG) (Sigma, UK.) was used as an immunogen for the mice. Each mouse received 70 or 100 μg of hIgG intraperitoneally.

### *Deaggregation of human IgG*

The hIgG was purchased in lyophilised form. After reconstitution in phosphate buffered saline pH 7.4 (PBS), it was found to contain 20-25% aggregates. These aggregates were pelleted by centrifugation at 150,000 *g* for 150 min (L8-70M, Beckman Instruments, Palo Alto, USA) and the 'deaggregated' contents of the top one-third of the centrifuge tube was then removed for use as an immunogen. The presence or absence of high molecular weight immunoglobulin aggregates was checked by SDS-polyacrylamide gel electrophoresis (SDS-PAGE), silver stained, and by fast protein liquid chromatography (FPLC) analysis (Pharmacia, Sweden). In further experiments, monomeric hIgG was isolated by Superose 12 and 6 (Pharmacia, Sweden) size exclusion chromatography columns connected on to an FPLC (Figure 1a, b). The monomeric fraction was used immediately after determination of concentration, in order to avoid reaggregation. SDS-PAGE analysis showed that after deaggregation by ultracentrifugation, a faint band at 300 kDa was visible; no such band was seen in the fraction purified by gel filtration.

### *Aggregation of human IgG*

Human IgG was heated for 2 h at 59°C and then left overnight on ice (Weigle, 1973). Additional aggregate formation was confirmed by FPLC analysis (Figure 1c). After determination of the protein concentration, the samples were stored at − 20°C.

**Figure 1** FPLC profile of human IgG (hIgG): **a**, after deaggregation using Superose 6 size exclusion chromatography; **b**, before deaggregation and **c**, after heat aggregation.

*Immunisation schedule*

Mice were given 70 μg or 100 μg of: the untreated, partially aggregated hIgG (ag. hIgG); the monomeric, deggregated (deag.hIgG) fraction; or the polymeric, aggregated (heat-ag.hIgG) fraction. Human IgG was administered i.p. in sterile PBS in a volume that never exceeded 0.8 ml. Control mice received an equal volume of sterile PBS alone. Some mice were killed and bled 10 days after the first injection of hIgG (to assay their primary immune response), others were challenged for a second time. Seven days later these animals were bled and their secondary response against hIgG was also measured. For each immunisation protocol, some mice also received cyclosporin A immunosuppression (detailed below). A minimum of three mice were used for each experimental situation.

*Immunosuppression with cyclosporin A*

Sandimmun, containing 100 mg ml$^{-1}$ cyclosporin A in oil, was obtained from Sandoz Pharmaceuticals (Switzerland). Cyclosporin A was administered to mice orally, throughout the immunisation period (10 days primary, 7 days secondary). Mice were given either 7 or 15 mg kg$^{-1}$ mouse$^{-1}$ daily. Control mice received vehicle (oil) alone.

*Patients*

Twenty-two patients with carcinoma of the ovary were given radiolabelled monoclonal antibody HMFG1 as part of their antitumour therapy. Patients received approximately 15 mg of radiolabelled antibody i.p. via a small catheter into the abdomen. A serum sample was taken before and 14 days after treatment.

*Monoclonal antibody HMFG1*

HMFG1 is a mouse IgG1 monoclonal antibody that recognises a large mucin-like molecule normally produced by the lactating breast, but also expressed by the majority (> 90%) of ovarian, breast and other cancers of epithelial origin (Taylor-Papadimitriou *et al.*, 1981). HMFG1 was radiolabelled with either iodine-131 (Amersham International, UK) using the *N*-bromo-succinamide (Sigma) method (Reay, 1982), or with yttrium-90 (AERE, Harwell, UK). Before yttrium-90 labelling, the antibody was conjugated to diethylenetriamine penta-acetic acid (DTPA) (Sigma) by the cyclic anhydrate method (Hnatowitch *et al.*, 1983). Yttrium-90 was then coupled to the antibody using a method based on that established for indium-111 (Hnatowitch *et al.*, 1983; Paik *et al.*, 1985).

*Assessment of aggregation of therapeutic monoclonal antibody preparations*

FPLC analysis and autoradiography of the HMFG1 before and 24 h after (serum sample) administration to the patients revealed that it was monomeric and that it remained monomeric after labelling with iodine-131 (Figure 2a). In contrast, yttrium-90-labelled HMFG1 was found to be partially aggregated (up to 40%) due to antibody cross-linkage via the bifunctional DTPA reagent (Figure 2b).

**Figure 2** FPLC profile of the mouse monoclonal antibody HMFG1 when labelled with: **a**, iodine-131; and **b**, yttrium-90.

*Measurement of the anti-immunoglobulin response*

All blood samples were left to clot overnight at 4°C. They were then centrifuged and the supernatant serum removed, aliquoted and stored at − 20°C. An enzyme-linked immunsorbent assay (ELISA) was used to measure the anti-Ig response. This has been described in detail elsewhere (Courtenay-Luck *et al.*, 1986). Briefly, 5 μg ml$^{-1}$ of either hIgG (to assay mouse sera) or HMFG1 (to assay patients' sera) was coated on to 96-well plates (Sterilin, UK) for 3 h at 37°C in bicarbonate buffer pH 9.6. After three washes with PBS containing 0.02% Tween 20 (Sigma) plates were incubated for 2 h at 37°C with serial 1:10 dilutions of test mouse or human sera, as appropriate. The plates were then washed with PBS/Tween as before, and a peroxidase-conjugated, species specific sheep anti-mouse Ig (mouse test sera) or species specific sheep anti-human Ig (human test sera) was applied (Amersham International). After incubation for 1 h at 37°C, the plates were washed as before and the substrate 2,2′-azino-di-[3-ethylbenzthiazolinesulphonate] (ABTS; Behring, FR Germany) was added. The development of colour in each well was measured by determining the absorbence at 405 nm using a Titertec multiscanner (Flow Laboratories, UK). All groups of sera were tested independently as well as pooled together. All data shown are from grouped sera. Where three sera for a group were not available, the data are shown as not tested (n.t.). Where a comparison between two or more ELISA plates was necessary, at least two appropriate reference sera were used. Data given are the absorbence values for serum samples diluted either 1:10 (Figures 3a and 5b, d, f) or 1:100 where there was a prozone effect at 1:10. Statistical analysis was performed using Student's *t* test. This was only used to compare data within, and not between, experiments. Each histogram presented represents a single experiment.

### Results

*Immune response to hIgG in BALB/c mice: effect of deaggregation by ultracentrifugation*

BALB/c mice were given 70 μg of polyclonal human IgG (in 0.8 ml sterile PBS) that was either untreated or had been deaggregated by ultracentrifugation, and the primary (10 days) response was measured. Control mice received 0.8 ml sterile PBS alone. A small difference was seen in the response of BALB/c mice to these hIgG preparations (Figure 3a, $P = 0.01$).

**Figure 3** **a**, Primary response of BALB/c mice to aggregated (ag.hIgG) and ultracentrifuge deaggregated (deag.hIgG) hIgG. Primary (**b**) and secondary (**c**) responses of BALB/c mice to aggregated (ag.), heat aggregated (heat-ag.) and chromatographically deaggregated (deag.) hIgG. All responses were measured by ELISA. The vertical axis shows absorbance at 405 nm. deag.X deag: primary immunization with deag.hIgG; secondary with deag.hIgG. deag.X ag.: primary immunization with deag.hIgG; secondary with ag.hIgG. ag.Xdeag.: primary immunization with ag.hIgG; secondary with deag.hIgG. ag.X ag.: primary immunization with ag.hIgG; secondary with ag.hIgG.

*Immune response to hIgG in BALB/c mice: effect of deaggregation by size exclusion chromatography*

BALB/c mice were given 100 μg of one of the following hIgG preparations (in 0.2 ml sterile PBS): untreated; deaggregated by Superose 12 size exclusion chromatography connected to an FPLC; or heat aggregated. Negative control mice received only 0.2 ml sterile PBS. The primary (10 day) immune response to these different immunogens is shown in Figure 3b. The untreated (ag.hIgG) and heat aggregated (heat-ag.hIgG) preparations were equally immunogenic. However, mice immunised with chromatographically deaggregated hIgG (deag.hIgG) showed a highly significant lowering of the immune response, in comparison to either ag.hIgG ($P = 0.0005$) or the heat-ag.hIgG ($P = 0.001$). Subsequently, all these groups were rechallenged with a further 100 μg of either deag.hIgG or ag.hIgG in all possible combinations of primary and secondary antigen. When tested 7 days later (secondary response) all groups that received at least one deag.hIgG immunisation were found to have responded significantly less than those that received two doses of ag.hIgG (Figure 3c). The $P$ values were 0.001 for ag.Xag. *vs* ag.Xdeag., 0.0021 for ag.Xag *vs* deag.Xag., and 0.001 for ag.Xag. *vs* deag.Xdeag.

*Immune response of CBA and C57 BL/6 mice to human IgG: effects of deaggregation using size exclusion chromatography*

One hundred μg of ag.hIgG, deag.hIgG or heat-ag.hIgG (0.2 ml in sterile PBS) was administered i.p. to CBA and C57BL/6 mice. The deag.hIgG was deaggregated using a Superose 6 size exclusion chromatography column connected to an FLPC. Control mice received 0.2 ml sterile PBS. The primary response to ag.hIgG and heat-ag.hIgG was high in CBA and C57BL/6 mice. In contrast, the response to deag.hIgG was very low in both strains of mice (Figure 4a,c), with $P < 0.008$ in all cases. On day 10 all groups were rechallenged with a further 100 μg of either deag.hIgG or ag.hIgG, and the secondary response was assayed 7 days later. All groups of mice showed a secondary response, although this varied in magnitude according to the immunisation schedule and the strain of mouse used (Figure 4b, d). In C57/BL6 mice the immune response was reduced when at least one injection was of the deaggregated material, either primary or secondary (Figure 4d, $P<0.05$). In contrast, for CBA mice, only those receiving deaggregated hIgG for both primary and secondary injections, showed a significantly reduced immune response (Figure 4b, $P<0.05$).

*Immune response to hIgG in BALB/c mice: effect of deaggregation by size exclusion chromatography in combination with cyclosporin A immunosuppression*

Human IgG was deaggregated by isolating the peak that corresponded to approximately 150 kDa using a Superose 6 size exclusion chromatography column connected to an FPLC (Figure 1a). BALB/c mice were immunised i.p. with 100 μg (in 0.2 ml sterile PBS) of either the untreated or the chromatographically deaggregated hIgG, or with PBS alone, and their primary response measured at day 10. Mice from each group were then boosted with a further 100 μg of either ag.hIgG or deag.IgG, and their secondary response assayed after 7 days. Throughout the 10-day primary immunisation period half the mice in each group received a daily oral dose of either 7 or 15 mg $kg^{-1}$ $day^{-1}$ cyclosporin A. Control mice received an equal volume of oil alone. The primary responses of these mice are summarised in Figure 5a, b. In contrast to the previous experiment, the mice responded equally well to both ag.hIgG and deag.hIgG. Cyclosporin A at 7 and 15 mg $kg^{-1}$ $day^{-1}$ reduced this primary response (Figure 5a, b), with $P < 0.0085$. Those mice that were to be assayed for the secondary response received cyclosporin A for the 7-day secondary immunisation period only. The data for the secondary response, assayed on day 17, are given in Figure 5c–f. As before, 15 mg $kg^{-1}$ $day^{-1}$ was sufficient to significantly

**Figure 4** Primary (**a**) and secondary (**b**) responses of CBA mice to aggregated (ag.hIgG), heat aggregated (heat-ag.hIgG) and chromatographically deaggregated hIgG (deag.hIgG). Primary (**c**) and secondary (**d**) responses of C57BL/6 mice to aggregated, heat aggregated and chromatographically deaggregated hIgG. All responses were measured by ELISA. The vertical axis shows absorbance at 405 nm. deag.X deag.: primary immunisation with deag.hIgG; secondary with deag.hIgG. deag.Xag.: primary immunisation with deag.hIgG; secondary with ag.hIgG. ag.X deag.: primary immunisation with ag.hIgG; secondary with deag.hIgG. ag.X ag.: primary immunisation with ag.hIgG; secondary with ag.hIgG.

reduce the mouse anti-hIgG response (Figure 5c, e; $P<0.002$), and we observed an additive effect with cyclosporin A and deag.hIgG (Figure 5c). Cyclosporin A at 7 mg $kg^{-1}$ $day^{-1}$ was insufficient to give immunosupression (Figure 5d, f) in all situations except that where the animals were both primed and boosted with deag.hIgG (Figure 5d; $P = 0.001$).

### *Immune response of patients with cancer to therapeutic mouse IgG monoclonal antibody: effect of deaggregation*

Twenty-two patients with ovarian cancer were given approximately 15 mg monoclonal antibody HMFG1 intraperitoneally, for therapeutic purposes. Eleven received antibody labelled with iodine-131 (80–150 mCi), while the other 11 received antibody labelled with yttrium-90 (5–20 mCi). All patients were receiving monoclonal antibody treatment for the first time. Each patient's anti-mouse IgG response was measured before and 14 days after therapy. The iodine-131 labelled monoclonal antibody was monomeric (Figure 2a), but the yttrium-90 labelled antibody contained up to 40% aggregates (Figure 2b), due to the DTPA coupling procedure. No significant difference was observed between the anti-mouse IgG response of these two groups (Figure 6).

### Discussion

One of the major problems that has to be overcome before monoclonal antibody therapy for cancer can reach its full potential is the immunogenicity of the administered xenogeneic immunoglobulin. Since 1962 when Dresser first reported that he could induce tolerance to bovine gammaglobulin many investigators have supported the view that deaggregated IgG is tolerogenic in some strains of mice ('susceptible') but not in others ('resistant') (Golub & Weigle, 1969; Lukic *et al.*, 1975*a, b*; Pepys & Taussig, 1974) The work presented in this paper was designed to investigate the possibility that deaggregation of the monoclonal antibodies used for *in vivo* therapy might render them tolerogenic to the recipient. We used two approaches. Firstly, we used a genetically 'inverted' experimental model in which we studied the immune response of mice to various preparations of human IgG to determine whether more stringent deaggregation could render IgG tolerogenic even to a 'resistant' animal. Secondly, we studied the immune responses of a series of patients with cancer to therapeutic mouse monoclonal antibody that was coupled with either iodine-131 (no aggregates) or yttrium-90 (contains aggregates).

The degree of deaggregation appears to be crucial in obtaining pure tolerogenic Ig monomers. In previous studies, careful deaggregation using $Na_2SO_4$ fractionation or biological filtration (Golub & Weigle, 1969; Lukic *et al.*, 1975*b*) was found to be more effective than ultracentrifugation (Dresser, 1962; Pepys & Taussig, 1974; Lukic *et al.*, 1975*b*; Das & Leskowitz, 1970; Benjamin *et al.*, 1986) for producing tolerogenic Ig. For this study we chose to deaggregate our IgG using FPLC gel filtration since this provides a rapid and highly efficient (>90% monomeric fraction recovered) method of deaggregation. A second important consideration is the method by which the anti-Ig response is detected. The main system used by previous investigators was the rate of biological clearance of iodine-125-labelled gammaglobulin (Golub & Weigle, 1969; Pepys & Taussig, 1974; Lukic *et al.*, 1975*b*). For the experiments presented here we have developed a highly sensitive ELISA to quantitate the anti-xenogeneic Ig response.

The induction of tolerance in mice to deaggregated IgG appears to be under genetic control. Thus, strains such as C57BL/6, A/J, DBA/2, CBA and C3H/HE mice have been shown to be 'susceptible' to the induction of unresponsiveness (Golub & Weigle, 1969; Lukic *et al.*, 1975*b*; Fujiwara & Cinader, 1974; Staples *et al.*, 1970), whereas BALB/c, SJL, NZB and DDD mice are particularly 'resistant' (Golub & Weigle, 1969; Lukic *et al.*, 1975*a*; Staples *et al.*, 1970; Hosono *et al.*, 1977; Playfair, 1971; Hosono & Fujiwara, 1979*a, b*). Our data support these findings to some extent, showing that while BALB/c are high responders, C57BL/6 and CBA are low responders to hIgG.

A comparison of the immune response that was generated

**Figure 5** Primary response of BALB/c mice to aggregated (ag.) and chromatographically deaggregated (deag.) hIgG, when 15 mg kg$^{-1}$ day$^{-1}$ (**a**) or 7 mg kg$^{-1}$ day$^{-1}$ (**b**) cyclosporin A (CSA) was orally administered. Secondary response of BALB/c mice to aggregated and deaggregated hIgG, when 15 mg kg$^{-1}$ day$^{-1}$ (**c,e**) or 7 mg kg$^{-1}$ day$^{-1}$ (**d, f**) cyclosporin A was orally administered. The response is measured by ELISA. The vertical axis shows absorbence at 405nm. deag.X[deag. + CSA]: primary immunisation with deag.hIgG; secondary with deag.hIgG in combination with CSA. deag.X[ag. + CSA]: primary immunisation with deag.hIgG; secondary with ag.hIgG in combination with CSA. ag.X[deag. + CSA]: primary immunisation with ag.hIgG; secondary with deag.hIgG in combination with CSA. ag.X[ag. + CSA]: primary immunisation with ag.hIgG; secondary with ag.hIgG in combination with CSA. deag.X deag: primary immunisation with deag.hIgG; secondary with deag.hIgG. deag.Xag.: primary immunisation with deag.hIgG; secondary with ag.hIgG. ag.X deag.: primary immunisation with ag.hIgG; secondary with deag.hIgG. ag.Xag.: primary immunisation with ag.hIgG; secondary with ag.hIgG.

**Figure 6** Anti-mouse Ig response of patients with cancer, who received either the deaggregated iodine-131 or the aggregated yttrium-90 labelled murine monoclonal antibody HMFG1. The levels were measured before (pre) and 14 days after (post) the therapeutic administration of the monoclonal antibody, by ELISA. Absorbence at 405nm is shown on the vertical axis.

to either aggregated or deaggregated hIgG prepared by FPLC showed that the chromatographically prepared monomers induced a smaller anti-hIgG response in both 'responder' and 'non-responder' strains of mice, although the level of reduction was less and more variable in the 'responder' BALB/c strain (ranging from 65% reduction, Figure 3b to no effect, Figure 5a, b). The data therefore indicate that deaggregation leads to a reduction in immunogenicity, but we have no evidence for the induction of tolerance.

Cyclosporin A reduces the primary and secondary B-cell immune responses, to both immunoglobulin preparations, presumably via its well known inhibitory action of T-cell activation and lymphokine secretion (Bickel *et al.*, 1987; Krusemeier & Snow, 1988; Borel *et al.*, 1977). Additional experiments showed that the secondary immune response to deaggregated hIgG, in combination with CSA, resulted in a depression of the immune response, that was greater than that achieved with either factor alone. Similar co-operative effects between CSA and deaggregation have recently been described in rabbits given murine monoclonal antibody (Ledermann *et al.*, 1988).

Parallel studies on the effects of Ig aggregation were carried out in 22 patients with cancer, who were receiving immunotherapy, where we could compare the immune response generated to non-aggregated (iodine-131 labelled) and aggregated (yttrium-90 labelled) mouse monoclonal antibody. However, in this situation both preparations were found to be equally immunogenic (Figure 6), despite the fact that the non-aggregated iodine-131 preparation was composed of pure monomers, as shown by FPLC (Figure 2) or SDS-PAGE analysis. These findings can probably be attributed to the fact that genetically controlled immune response differences would be difficult to see in the outbred human population.

Alternatively, it is possible that we are underestimating the immune response to aggregated murine Ig since yttrium-90-labelled monoclonal antibodies result in higher bone marrow toxicity (and hence immunosuppression) than those labelled with iodine-131 (unpublished observations). A further contributory factor in our inability to induce tolerance to monomeric murine Ig in patients may be the fact that the therapeutic antibody has specificity for a cell surface antigen, since such reagents are less tolerogenic than those that either bind to soluble antigen or have no specific target antigen in the treated recipient (as is the case with our mice given polyclonal hIgG) (Benjamin et al., 1986).

The route of administration is also thought to influence the ability to induce suppression of an immune response. Successful tolerance has been achieved with both intravenous (i.v.) and intraperitoneal (i.p.) routes (Golub & Weigle, 1969; Pepys & Taussig, 1974; Lukic et al., 1975b). We selected i.p. administration for our studies since this is the regional administration route used to optimise the localisation of therapeutic antibodies in our patients with ovarian cancer (Epenetos et al., 1987). It is possible that an i.v. route might be more effective in generating immune unresponsiveness in these patients.

Our studies indicate that deaggregation alone is not sufficient to abrogate the human anti-mouse Ig response in patients with cancer undergoing monoclonal antibody therapy. The concommitant use of deaggregated immunoglobulin and cyclosporin A immunosuppression might be expected to have an additive effect in reducing the immune response, although the bone marrow toxicity and generalised immunosuppression resulting from cyclosporin A therapy make this approach less attractive. Our goal must therefore still remain the induction of tolerance that is antigen-specific, leaving the rest of the immune system intact.

We thank Dr J. Taylor-Papadimitriou for the HMFG1 monoclonal antibody, Dr N.S. Courtenay-Luck, Miss D. Snook and Mr B. Dhokia for their help with the iodine-131 and yttrium-90 radiolabelling, and Mrs M. Bowe and Miss R. Parks for the excellent secretarial assistance. This research was funded by the Cancer Research Campaign.

### References

BENJAMIN, R.J., COBBOLD, S.P., CLARK, M.R. & WALDMAN, H. (1986). Tolerance to rat monoclonal antibodies. Implications for serotheraphy. *J. Exp. Med.*, **163**, 1539.

BICKEL, M., TSUDA, H., AMSTAD, P. and 4 others (1987). Differential regulation of colony-stimulating factors and interleucin 2 production by Cyclosporin A. *Proc. Natl Acad. Sci. USA*, **84**, 3274.

BOREL, J.F., FEURER, C., MAGNEE, C. & STAHELIN, H. (1977). Effects of the new anti-lymphocytic peptide Cyclosporin A in animals. *Immunology*, **32**, 1017.

CARRASQUILLO, J.A., BUNN, P.A., Jr., KEENAN, A.M. and 12 others (1986). Radioimmunodetection of cutaneous T-cell lymphoma with 111-In-labelled T101 monoclonal antibody. *N. Engl. J. Med.*, **315**, 673.

CHATAL, J.F., SACCAVINI, J.C., FUMOLEAU, P. and 5 others (1984). Immunoscintigraphy of colon carcinoma. *J. Nucl. Med.*, **25**, 307.

COURTENAY-LUCK, N.S., EPENETOS, A.A., MOORE, R. and 4 others (1986). Development of primary and secondary immune responses to mouse monoclonal antibodies used in the diagnosis and therapy of malignant neoplasms. *Cancer Res.*, **46**, 6489.

DAS, S. & LESKOWITZ, S. (1970). The kinetics of *in vivo* tolerance induction in mice. *J. Immunol.*, **105**, 938.

DRESSER, D.A. (1962). Specific inhibition of antibody production. II paralysis induced in adult mice by small quantities of protein antigens. *Immunology*, **5**, 378.

EPENETOS, A.A., MUNRO, A.J., STEWART, A. and 14 others (1987). Antibody-guided irradiation of advanced ovarian cancer with intraperitoneally administered radiolabelled monoclonal antibodies. *J. Clin. Oncol.*, **5**, 1890.

FUJIWARA, M. & CINADER, B. (1974). Cellular aspects of tolerance. III. The responsiveness of T-cells from tolerant donors after exposure to a cross-reactivity antigen. *Cell. Immunol.*, **12**, 1.

GOLUB, E.S. & WEIGLE, W.O. (1969). Studies on the induction of immunologic unresponsiveness. III. Antigen form and mouse strain variation. *J. Immunol.*, **102**, 389.

HNATOWITCH, D.T., CHILDS, R.L., LANTEIGNE, D. & NAJAFI, A. (1983). The preparation of DTPA-coupled antibodies radiolabelled with metalilic radionuclides: an improved method. *J. Immunol. Meth.*, **65**, 147.

HOSONO, M., CINADER, B. & ELLERSON, J. (1977). Tolerance induction as an index of age-related changes. *Immunol. Commun.*, **6**, 237.

HOSONO, M. & FUJIWARA, M. (1979*a*). Studies on the resistance to tolerance induction against human IgG in DDD mice. I. Organ differences of tolerogen susceptibility and cellular sites responsible for the resistance. *Cell. Immunol.*, **42**, 279.

HOSONO, M. & FUJIWARA, M. (1979*b*). Studies on the resistance to tolerance induction against human IgG in DDD mice. II. Tolerogen resistant T-cell population in the spleen. *Immunology*, **37**, 353.

KRUSEMEIER, M. & SNOW, C.E. (1988). Induction of lymphokine responsiveness of hapten-specific B lymphocytes promoted through an antigen-mediated T helper lymphocyte interaction. *J. Immunol.*, **2**, 367.

LEDERMANN, J.A., BEGENT, R.H.J. & BAGSHAWE, K.D. (1988). Cyclosporin A prevents the anti-murine antibody response to a monoclonal anti-tumour antibody in rabbits. *Br. J. Cancer*, **58**, 562.

LUKIC, M.L. & LESKOWITZ, S. (1974). Tolerance induction with bovine gamma-globulin in mouse radiation chimaeras depends on macrophages. *Nature*, **252**, 605.

LUKIC, M.L. WORTIS, H.H. & LESKOWITZ, S. (1975*a*). A gene locus affecting tolerance to BGG in mice. *Cell. Immunol.*, **15**, 457.

LUKIC, M.L., COWING, C. & LESKOWITZ, S. (1975*b*). Strain differences in ease of tolerance induction to bovine gamma-globulin: dependence on macrophage function. *J. Immunol.*, **114**, 503.

PAIK, C.H., HONG, J.J., EBBERT, M.A., HEALD, S.C., REBA, R.C. & ECKELMAN, W.C. (1985). Relative reactivity of DTPA, immunoreactive Ab-DTPA conjugates, non-immunoreactive Ab-DPTA conjugates toward Indium-111. *J. Nucl. Med.*, **26**, 482.

PEPYS, M.B. & TAUSSIG, M.J. (1974). Complement-independence of tolerance induction. *Eur. J. Immunol.*, **4**, 349.

PLAYFAIR, J.H.L. (1971). Strain differences in the immune responses of mice. 3. A raised tolerance threshold in NZB thymus cells. *Immunology*, **21**, 1037.

REAY, P. (1982). Use of N-bromo-succinamide for the iodination of proteins for radioimmunoassay. *Ann. Clin. Biochem.*, **19**, 129.

SCHROFF, R.W., FOON, K.A., BEATTY, S.M., OLDHAM, R.K. & MORGAN, A.C. JR (1985). Human anti-murine immunoglobuln responses in patients receiving monoclonal antibody therapy. *Cancer Res.*, **45**, 879.

STAPLES, D.J., STEINBERG, A.D. & TALAL, N. (1970). Induction of immunological tolerance in older New Zealand mice, repopulated with young spleen, bone marrow and thymus. *J. Exp. Med.*, **131**, 1223.

TAYLOR-PAPADIMITRIOU, J., PETERSON, J.A., ARKLIE, J., BURCHELL, J., CERIANI, R.L. & BODMER, W.F. (1981). Monoclonal antibodies to epithelium specific components of the human milk fat globule membrane: production and reaction with cells in culture. *Int. J. Cancer*, **28**, 17.

WEIGLE, W.O. (1973). Immunological unresponsiveness. *Adv. Immunol.*, **16**, 61.