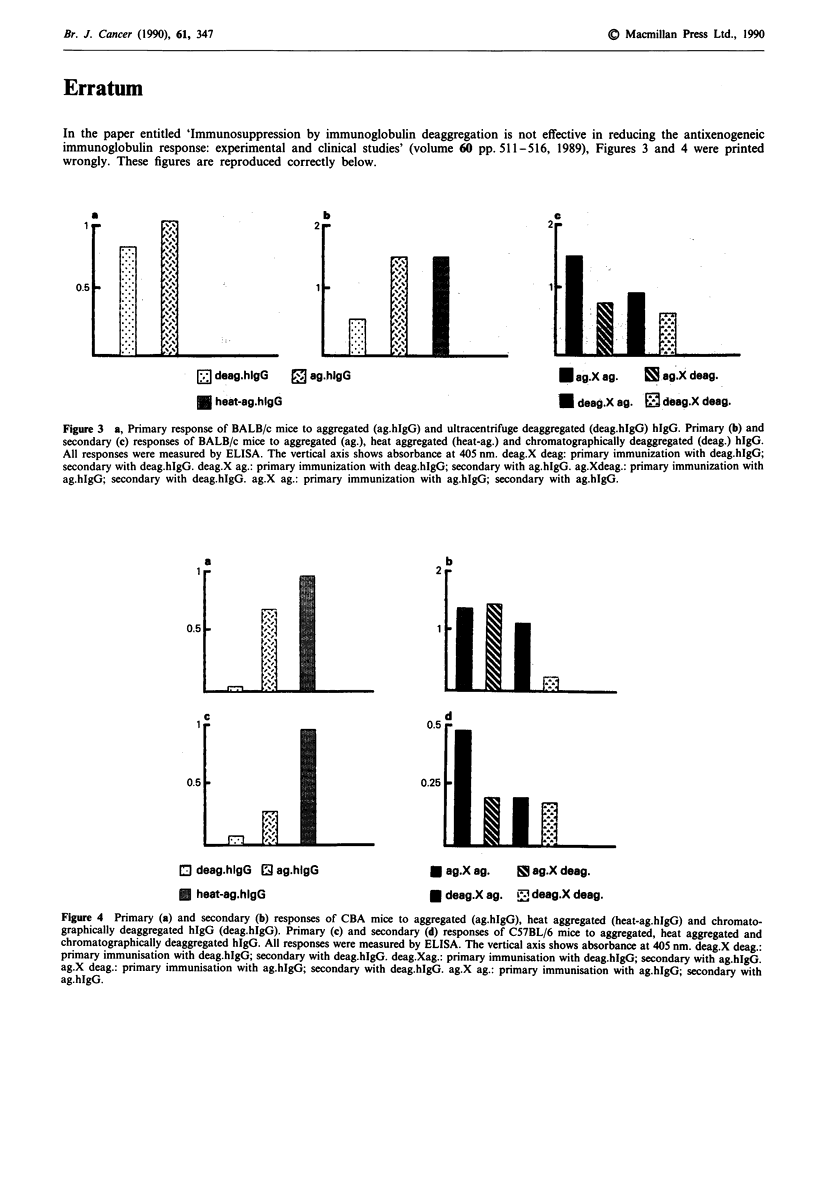# Erratum

**Published:** 1990-02

**Authors:** 


 © Macmillan Press Ltd., 1990

### Erratum

In the paper entitled 'Immunosuppression by immunoglobulin deaggregation is not effective in reducing the antixenogeneic immunoglobulin response: experimental and clinical studies' (volume 60 pp. 511–516, 1989), Figures 3 and 4 were printed wrongly. These figures are reproduced correctly below.

**Figure 3** **a**, Primary response of BALB/c mice to aggregated (ag.hIgG) and ultracentrifuge deaggregated (deag.hIgG) hIgG. Primary (**b**) and secondary (**c**) responses of BALB/c mice to aggregated (ag.), heat aggregated (heat-ag.) and chromatographically deaggregated (deag.) hIgG. All responses were measured by ELISA. The vertical axis shows absorbance at 405 nm. deag.X deag: primary immunization with deag.hIgG; secondary with deag.hIgG. deag.X ag.: primary immunization with deag.hIgG; secondary with ag.hIgG. ag.Xdeag.: primary immunization with ag.hIgG; secondary with deag.hIgG. ag.X ag.: primary immunization with ag.hIgG; secondary with ag.hIgG.

**Figure 4** Primary (**a**) and secondary (**b**) responses of CBA mice to aggregated (ag.hIgG), heat aggregated (heat-ag.hIgG) and chromatographically deaggregated hIgG (deag.hIgG). Primary (**c**) and secondary (**d**) responses of C57BL/6 mice to aggregated, heat aggregated and chromatographically deaggregated hIgG. All responses were measured by ELISA. The vertical axis shows absorbance at 405 nm. deag.X deag.: primary immunisation with deag.hIgG; secondary with deag.hIgG. deag.Xag.: primary immunisation with deag.hIgG; secondary with ag.hIgG. ag.X deag.: primary immunisation with ag.hIgG; secondary with deag.hIgG. ag.X ag.: primary immunisation with ag.hIgG; secondary with ag.hIgG.